# Attitudes of Swedish Language Twitter Users Toward COVID-19 Vaccination: Exploratory Qualitative Study

**DOI:** 10.2196/42357

**Published:** 2023-02-22

**Authors:** Safwat Beirakdar, Leon Klingborg, Sibylle Herzig van Wees

**Affiliations:** 1 Karolinska Institute Department of Global Public Health Stockholm Sweden

**Keywords:** COVID-19, vaccine hesitancy, COVID-19 vaccines, social media, Twitter, qualitative analysis, World Health Organization, WHO’s 3C model

## Abstract

**Background:**

Social media have played an important role in shaping COVID-19 vaccine choices during the pandemic. Understanding people’s attitudes toward the vaccine as expressed on social media can help address the concerns of vaccine-hesitant individuals.

**Objective:**

The aim of this study was to understand the attitudes of Swedish-speaking Twitter users toward COVID-19 vaccines.

**Methods:**

This was an exploratory qualitative study that used a social media–listening approach. Between January and March 2022, a total of 2877 publicly available tweets in Swedish were systematically extracted from Twitter. A deductive thematic analysis was conducted using the World Health Organization’s 3C model (*confidence*, *complacency*, and *convenience*).

**Results:**

*Confidence* in the safety and effectiveness of the COVID-19 vaccine appeared to be a major concern expressed on Twitter. Unclear governmental strategies in managing the pandemic in Sweden and the belief in conspiracy theories have further influenced negative attitudes toward vaccines. *Complacency*—the perceived risk of COVID-19 was low and booster vaccination was unnecessary; many expressed trust in natural immunity. *Convenience*—in terms of accessing the right information and the vaccine—highlighted a knowledge gap about the benefits and necessity of the vaccine, as well as complaints about the quality of vaccination services.

**Conclusions:**

Swedish-speaking Twitter users in this study had negative attitudes toward COVID-19 vaccines, particularly booster vaccines. We identified attitudes toward vaccines and misinformation, indicating that social media monitoring can help policy makers respond by developing proactive health communication interventions.

## Introduction

### Background

Sweden was seriously affected by the pandemic compared with some European countries. In relation to neighboring Nordic countries, Sweden has had the highest number of infected and terminally ill patients, with 2,500,000 positive cases and >16,000 deaths as of February 2022 [1,2].

The Public Health Agency of Sweden implemented various interventions and strategies to speed up COVID-19 vaccine uptake, including media campaigns to promote vaccination and facilitate vaccination accessibility across the Stockholm region through mobile vaccination buses [3]. However, vaccine hesitancy among the public arguably slowed down the vaccination process, and a small percentage of the public is still showing reluctance to COVID-19 vaccination in general and boosters in particular [4]. According to the Public Health Agency of Sweden, 86.4% of the population received 2 doses of the COVID-19 vaccine. Uptake of the third vaccine has been lower, and only 66.6% of the Swedish population who are eligible for a third vaccine have been vaccinated [5]. Although these numbers are not alarming in comparison with other countries, Sweden has in the past few years encountered persistent vaccine hesitancy and the circulation of rumors about vaccines in certain migrant communities, in communities that hold fringe political views, and in anthroposophic communities [6].

Globally, skepticism about vaccine effectiveness and safety has been a consistent challenge, and the rise in vaccine hesitancy has become an urgent concern and one of the top 10 threats to global health in 2019, according to the World Health Organization (WHO) [7]. Several factors contribute to the personal decision to take the vaccine, but social media have played an important role in promoting vaccine scarcity. Social media accelerated the spread of misinformation by providing a platform for vaccine-hesitant communities to spread rumors, ultimately shaking public trust in the COVID-19 vaccine [8,9].

Since the beginning of the COVID-19 pandemic, myths and rumors have circulated on social media regarding the virus’s origin, spread, symptoms, severity, treatments, and the safety and effectiveness of its vaccines [10]. Some of these rumors include concerns about the safety of COVID-19 vaccines because of their rapid development and the use of the novel concept of messenger RNA, which some claimed causes infertility [11]. Furthermore, conspiracy theories linking the spread of the virus to 5G mobile technology and implanted microchips have been prominent on social media [10]. In Sweden, there is little information about what rumors circulate in the Swedish language on social media.

The WHO defines false information that systematically spreads in time of disease outbreaks as *infodemics* [12]. Infodemics constitute the rapid proliferation of harmful messages through social media platforms, causing confusion and mistrust among the public [12]. For instance, social media platforms played an important role in polarizing the public against the human papillomavirus vaccination in Japan in 2013 [13], where negative media campaigns overtook the scientific evidence provided by local authorities, leading to a decline in vaccine uptake to less than 1% [13]. Another incident was seen in Denmark, where public confidence in the human papillomavirus vaccination dropped significantly after the spread of a documentary based on teenagers’ experiences with complications after getting vaccinated [14]. Moreover, increasing evidence suggests that negative vaccine posts on social media contribute to vaccine hesitancy by altering the risk perceptions of individuals [15]. An experimental study by Betsch et al [15] demonstrated that 5-10 minutes of exposure to such materials is sufficient to trigger negative attitudes about vaccination. Similarly, a study on the uptake of the influenza vaccine showed that uptake was lower among people who were exposed to misinformation distributed on the internet [16]. Given the growing concern over fading confidence in the COVID-19 vaccine and as little knowledge is available on rumors and misinformation in Sweden, this study examines potential traces of *infodemics* that are at play in the Swedish Twitter discourse about COVID-19 vaccines.

### Objective

The European Center for Disease Prevention and Control published a report in June 2021 that encouraged member states to gain a better understanding of the misinformation landscape on social media [17]. In Sweden, several surveys have been conducted by the Public Health Agency of Sweden in the past 2 years to measure the public acceptance of COVID-19 vaccines. However, to date, no study has been published on the public discourse found on social media platforms, such as Twitter, where people express their opinions without probing from researchers. Previous literature has focused more on immigrants,such as those from the Somali community in Stockholm and the anthroposophic communities [6], as these 2 groups have shown a pattern of vaccine hesitancy [6]. Hence, there is a gap in the literature in exploring vaccination concerns and rumors among people who are active on social media in Sweden, and this study aimed to contribute to this knowledge.

## Methods

### Study Design

This was an exploratory qualitative study that used a social media–listening approach. Data from Twitter were gathered using Netlytic, a wrapper for the Twitter application programming interface (API; version 1), and Boolean operator search queries. Qualitative deductive thematic data analysis was guided by the WHO’s 3C model: confidence, complacency, and convenience. The WHO’s 3C model classifies the factors influencing vaccine hesitancy in individuals or groups into 3 main categories: confidence, complacency, and convenience [18,19]. Confidence refers to both trust in the effectiveness and safety of the vaccine and trust in governmental policies and motivation behind recommending the vaccine [7]. Complacency is related to the level of risk that individuals perceive in terms of becoming infected with the disease, thereby shaping their personal belief in the necessity of vaccination [7]. Finally, convenience refers to the availability and accessibility of vaccination and is also related to the quality of vaccination services [7].

Twitter was selected as the main data source because it is an important social media platform for disseminating information and sharing opinions [20]. It is a popular and trusted source used by many governmental agencies, political leaders, and famous influencers to address and interact with the public [21]. In addition, compared with other social media platforms, Twitter provides greater access to data and the ability to retrieve real-time data [22]. Twitter allows users to post pictures, videos, and “tweets” that constitute short texts with a maximum of 280 characters [22], and users interact with each other and engage in conversations using the like, reply, and retweet features [20].

### Data Extraction

The study analyzed public attitudes by reviewing tweets posted in Swedish. This was accomplished through “Netlytic,” a web-based service that allows for real-time data scraping from various social media platforms that publish publicly available posts [23,24]. Specifically, this study used Netlytic’s wrapper and interface for the Twitter API. Netlytic has been used in multiple social media–listening studies [24]. The parameters in Netlytic were set to capture tweets in Swedish using complex search queries linked with Boolean operators (OR and AND), instead of single terms and hashtags, to obtain more relevant tweets when retrieving data from the Twitter API [22].

Boolean search queries were set based on the most-used hashtags and terms in Sweden regarding COVID-19 according to Google Trends, Statista, and Twitter [25]. The final search queries are presented in Textbox 1. Only neutral and general terms were used to avoid skewing the data and influencing results.

To reduce duplicates, Netlytic was set to exclude retweets while importing data. The study did not filter according to geographical location, as many users chose to hide their location for privacy concerns. Data scraping was scheduled to run the search queries weekly to match the Netlytic settings, because tweets older than 1 week would not be captured [23].

The data scraping covered 2 months from January 24, 2022, to March 24, 2022. The timeline reflected an important period of many changes, including the start of the booster shot recommendation [26] and the dominance of “omicron,” a new variant that is highly transmissible and less susceptible to vaccines [27,28]. In addition, by February 9, 2022, Sweden entered a new phase of the pandemic, where all restrictions implemented to control the virus were removed [29].

**Boolean search queries used in the study.**

(Corona OR covid OR coronaviruset OR coronavirussverige OR coronasverige OR coronavirus OR COVID-19) AND (spruta OR vaccin OR coronavaccin OR vaccinspruta OR coronaspruta OR påfyllnadsdos OR tredjedos)

### Sample Size

**Figure 1:**
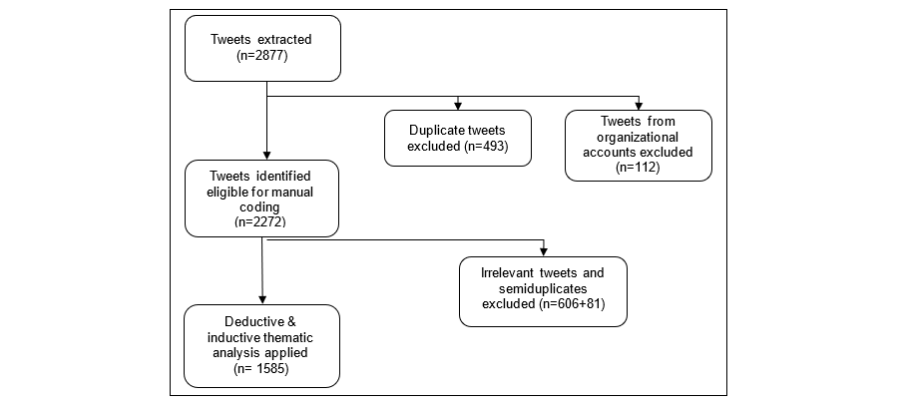
Tweet selection flow.

All tweets from scraping iterations were merged into a single data set. The total number of retrieved tweets was 2877, which underwent cleaning and eligibility screening phases (Figure 1).

The master sheet was cleaned from duplicate tweets (n=493), which included copy-pasted text with no changes. In addition, as the study aimed to explore individuals’ attitudes, tweets from organizational accounts (n=112), such as RegionStockholm, Krisinformation, WHO, Public Health Agency of Sweden, and Dagensnyheter, were removed from the data set.

Account names were also removed from the data set for ethical considerations.

The eligibility screening phase was conducted using the qualitative data analysis software NVivo (version 12 Pro; QSR International) with 2272 tweets. Tweets that did not present personal opinions about COVID-19 vaccines, were irrelevant to the research topic, or contained unclear statements were coded as irrelevant and excluded from the study (n=606). Irrelevant posts were predominantly posts that did not present personal opinions (including news, posts from organizations, and advertisements), and there were a few posts that were not included because the statement was not legible. In addition, semiduplicated tweets that included changes but did not present additional context compared with their original tweets were also excluded (n=81). As a result, tweets that contained a clear attitude related to the COVID-19 vaccines—whether the tweets were in favor of vaccination or skeptical toward it—were eligible for the qualitative analysis (n=1585). All tweets found eligible (n=1585) were included in the final sample.

### Data Analysis

The data were analyzed using qualitative thematic analysis (TA), inspired by the 2006 guide by Braun and Clarke [30]. TA was chosen because of its flexibility in answering research questions [30]. Moreover, TA is suitable for large amounts of data, as it provides a rich and inclusive analysis by reflecting the nuances within the data [30,31].

The TA was also led by the 3C model: confidence, complacency, and convenience [4]. This model has been used in many vaccine studies to understand the factors that influence vaccination [32,33]. Qualitative coding was conducted blindly by 2 individual coders (SB and SHvW) using NVivo 12. A mixture of deductive and inductive qualitative methods was applied by adopting TA according to Braun and Clarke [30,34]. This hybrid approach allowed for the flexibility of creating categories that emerged from the data rather than relying solely on the 3C framework. The data set was reviewed to gain familiarity with the data. Next, the themes were deductively predetermined, and the 3C of confidence, complacency, and convenience were set in NVivo. Subsequently, the tweets were inductively coded according to their meanings within the corresponding themes. For each theme, tweets were organized within categories according to emerging patterns. Themes and categories were not mutually exclusive; however, a tweet could be coded into one or more themes or categories. The themes’ titles were adjusted according to the findings. The resulting coding tree was discussed and agreed between the two coders.

### Trustworthiness

To ensure the trustworthiness of the qualitative research process, we applied the following strategies [35]: First, we applied clear criteria for the purposive sampling strategy. Second, the coding was completed by 2 researchers blindly. We also applied the overall peer scrutiny of the research project, whereby the research team regularly discussed emerging research challenges. This included reflection on their own backgrounds, which may lead to bias [35].

### Ethical Considerations

No ethics approvals were needed as the study analyzed publicly available data on the internet. All tweets identified in the study were anonymized after screening for eligibility to protect the privacy of users. Aspects of confidentiality and anonymization of data were respected as no data used in the final report can be linked to actual users. One of the measures taken to protect the identity of Twitter users behind the tweets in our data set was to translate all the quotes used in the analysis, ensuring that they cannot be traced back to their author. In addition, no interaction occurred between the study researchers and Twitter users. This approach is consistent with guidelines on the ethical conduct of qualitative research in web-based communities [36,37].

## Results

### Overview of Themes

The analysis resulted in 3 main themes and 18 categories, guided by the WHO’s 3C model (Textbox 2). The themes included confidence—safety and effectiveness concerns and mistrust in authorities; complacency—fading belief in vaccination necessity; and convenience—unappealing vaccination services and unclear information. In this section, each theme is described, and selective quotes are used to present the categories.

**Study results.**

**Themes and categories**

- Confidence: safety and effectiveness concerns and mistrust in authorities
  Concerns about messenger RNA COVID-19 vaccines safety and side effects
  COVID-19 vaccines effectiveness is limited
  The risks from the vaccines outweigh the benefit
  Concerns about the number of booster shots
  The spread of rumors and conspiracy theories
  Lack of transparency from the government and the Public Health Agency of Sweden
  Limited trust in the authority’s management of the COVID-19 pandemic
  Against mandatory vaccination and vaccine passes
  The media presented biased evidence in favor of COVID-19 vaccination
  Mistrust in scientific experts and pharmaceutical companies
- Complacency: fading belief in vaccination necessity
  The perceived necessity of vaccination against COVID-19 is low, especially among healthy adults
  Changes in the perceived risk of COVID-19 infection
  Natural immunity is superior to vaccines
  Conflicting opinions toward children’s vaccination against COVID-19
- Convenience: unappealing vaccination services and unclear information
  Limited availability of COVID-19 vaccination appointments
  COVID-19 vaccination services are unorganized
  Contradicting evidence
  Unanswered questions related to COVID-19 vaccination

### Confidence: Safety and Effectiveness Concerns and Mistrust in Authorities

The analysis shows that *confidence* is an important barrier to COVID-19 vaccination uptake.

#### Safety and Effectiveness Concerns

The analysis demonstrated that there are multiple safety concerns related to COVID-19 vaccines. There is a shared belief that the messenger RNA vaccines are produced too quickly and do not undergo all the testing processes required for approval. This rapid development of vaccines has resulted in side effects:

It takes several years to develop and test vaccines before they are released on the market. The C-vax [COVID-19 vaccines] is quickly developed and emergency-approved in just a few months. Therefore, more people get side effects than COVID.

In addition to the number of side effects, there was a specific concern regarding the severity of side effects and deaths related to vaccination:

COVID-19 injections have probably caused 2-50 million deaths and many more disabling injuries worldwide.

In addition, some tweets compared the number and severity of COVID-19 vaccines’ side effects to the H1N1 pandemic influenza vaccine Pandemrix, for example:

Side-effects reported by the Medical Products Agency. Right now, just over 95,000 are reported, just over 17,000 handled and just over 9,000 treated as serious. Compare with Pandemrix which had 300 severe, narcolepsy.

In terms of effectiveness, the data show that Twitter users in this study perceived vaccines as prophylactic injections, which reduce the severity of the infection. However, they did not consider them as effective as traditional vaccines. Many tweets expressed people’s frustration with becoming infected after being vaccinated:

Vaccines usually prevent diseases, right? At least the ones I have taken from birth onwards. The current COVID-19 “vaccine” is useless.

The skepticism and concerns about the vaccines seen in the data were primarily related to COVID-19 vaccines, as many tweets clearly expressed trust in other vaccines:

Being against the COVID-19 vaccine does not mean that you are against all vaccines.

However, some people expressed mistrust of future vaccines:

The failure of the COVID-19 vaccine makes me hesitant about any future vaccine.

Moreover, many people argued that the risk of vaccination outweighs its benefits:

There’s no way I’m taking a third Covid-19 syringe! The side effects after both make me give up. In addition, my daughter and her husband were sick, really sick, despite two syringes! So no thanks!

Several tweets reported safety concerns regarding the number of injections the body can tolerate and the immunity period provided by the vaccines, which are continuously decreasing. There were many sarcastic tweets on the booster shots:

You need to take booster shots until you are dead.

To add to the uncertainty about effectiveness, there were repeated rumors, myths, and misinformation about the COVID-19 vaccine, for example, that COVID-19 vaccines cause AIDS by weakening the immune system of the human body:

The more “vaccines” a person receives against the COVID-19 coronavirus, the faster the body will die from the AIDS-like immune loss syndrome!

Moreover, theories regarding the origin of the virus are rampant. Some argue that the virus was synthesized by political forces and that COVID-19 vaccines are biological weapons used against the public:

The virus came from a lab in Wuhan that was sponsored by the US government to conduct “gain-of-function” research that was banned by Obama.

#### Mistrust in Authorities

The data show that there was mistrust in the statistics and numbers related to the COVID-19 vaccines’ side effects and infection rates published by the authorities. For example, it was highlighted that the Public Health Agency of Sweden did not report the full numbers to encourage vaccination:

The problem is generally that the numbers are inflated and unreliable...Why not report who died OF COVID? Why are patients with syringes 1 + 2 reported as unvaccinated? Why not report figures for the unvaccinated?

The tweets in this study show that Sweden’s regulation was inconsistent with those of other countries. In addition, many tweets criticized the government’s delay in taking action, which led to serious consequences. For example, they accused the government of not protecting older adults:

Sweden’s strategy can never be “right.” A choice was made, in February 2020, where it was decided that it was ok to let the elderly get sick and die before they knew how to cure COVID-19 or have a vaccine. It is morally indefensible.

Furthermore, Twitter users in this study expressed skepticism based on governmental recommendations concerning COVID-19 vaccines:

On 12 January, the Swedish Public Health Agency stated that the vaccine protects well against serious illness, also against the omicron variant, for more than 6 months. That was less than THREE weeks ago! They currently have no idea what they are doing.

The enforcement of the vaccine pass was further criticized. The tweets expressed their disapproval of mandatory vaccination, even for people who took the vaccine, as it was perceived as a violation of personal freedom:

I have taken two doses of the vaccine and became ill with corona. The vaccine pass does not reduce the spread of infection, it is only a way to control people.

Moreover, the media agencies were criticized for being biased toward the government, where they blindly supported governmental decisions and undermined space for critical opinions.

The last category within this theme is the mistrust of scientific experts and pharmaceutical companies. The data demonstrate that there was a common belief that pharmaceutical companies benefit the most from the sale of vaccines. Some tweets suggested that scientific experts were pushed to ignore other potential factors to promote vaccination:

Everything that was not done to prevent the spread of COVID-19 that could have worked, ivermectin [ivermectin is an antiparasitic drug used by some countries to treat COVID-19], Vitamin D, etc. Instead, they all invested in one card—vaccination with a vaccine they had not tried before.

### Complacency: Fading Belief in Vaccination Necessity

Tweets analyzed under the *complacency* theme showed that young healthy adults felt they were not at risk. Instead, Twitter users in this study believed that only the older adults and people with chronic diseases were at high risk of hospitalization:

No one under the age of 50 would have become sicker without a vaccine. It would most likely have been just as mild symptoms anyway. Greater risk of crossing the street than getting seriously ill in COVID-19 if you are healthy and younger.

Many studies have compared COVID-19 infection with the usual influenza infection. In addition, the analysis of the tweets highlights that the new mild variants negatively affect people’s willingness to be vaccinated, even among infected individuals who reported strong symptoms:

Now COVID-19 is like a severe cold, I had Omicron now, had pain in the body, a little runny nose, sore throat, headache where I thought the eyes would fall out a little awkwardly with asthma but always so with a cold. I did not need a vaccine for this.

Moreover, there was a common belief that direct infection provides better protection than the immunity provided by vaccines. Many tweets expressed that individuals would rather be infected with COVID-19 than get vaccinated:

No one who has had COVID-19 needs to be vaccinated, natural immunity is superior to the temporary protection that this vaccine provides.

Swedish Twitter users in this study expressed a strong trust in natural immunity; therefore, many tweets encouraged other people to get infected to be protected:

Omicron is just a vaccine without a reservation

for a time slot for vaccination

Conflicting opinions were observed among the retrieved tweets on the necessity of vaccinating children against COVID-19, where the same arguments used to demand vaccination for children were used against vaccination but in a different context. Many tweets argued that children were also at risk of contracting COVID-19, contrary to what was previously believed. Tweets in favor of vaccination highlight that it is a child’s right to get vaccinated and be provided with the best possible care:

COVID-19 is to be spread and children between 5-11 years are deprived of the opportunity to be protected with a vaccine.

In addition, some Twitter users expressed fear for their children, as they can become severely ill and require hospitalization:

Many children are cared for in COVID-19 hospitals. The fact that they are offered vaccines is important to reduce the risk of them being seriously affected.

By contrast, tweets against vaccinating children expressed disbelief in the other group’s evidence, where they insisted that there is certainly no reason to fear COVID-19 infection:

COVID-19 is not dangerous for children. This is just propaganda because they want to throw vaccines at everyone when they have invested so much in it.

Similar to what was found in terms of the low perceived risk of COVID-19 in healthy adults, many people expressed that the risk of becoming seriously ill among healthy children is relatively low:

There is still no reason at all to vaccinate healthy children. Children who have risk factors are another matter, but there is in principle no healthy child in the whole world who has died from COVID-19 during the latter part of the pandemic.

### Convenience: Unappealing Vaccination Services and Unclear Information

The *convenience* theme revealed that some tweets discussed unappealing vaccination services and unclear health information. The analysis showed that there were complaints regarding the limited availability of vacant slots:

I’m unvaccinated, my wife had COVID-19 last week. It was a severe flu with a high fever for a few days...I was going to get vaccinated, but it was hard to find anything near where I live.

Available vaccination appointments were especially a problem for booster shots, and those who managed to get vaccinated complained of long waiting queues:

Today I took the 3rd vaccine against COVID-19. Cheers to us who stood in line for about an hour.

Moreover, some tweets revealed dissatisfaction with the vaccination system, as they did not receive invitations for their doses according to the published guidelines:

Tested the phone booking and seemed to be free to come forward. Most people wonder why I did not receive an offer...According to 1177 [Swedish health information website and number], those who received the second dose in mid-August will receive an offer today, I received it at the end of July.

In addition, many people on Twitter felt lost while following contradicting evidence and information related to COVID-19 vaccines distributed on the internet. Many people have highlighted their limited ability to understand scientific reports:

I think people have a hard time understanding that what is coming out here is true. There are research articles that claim completely different things, so it is not surprising that people get confused.

Many Twitter users felt that they needed more clarification regarding the COVID-19 vaccines. Tweets described insufficient information on the effects and immunity provided by the COVID-19 vaccines.

Why would it make sense to get vaccinated when you have had COVID-19? Why not highlight the risks of vaccines as well as the benefits?

In addition, many questions were related to the COVID-19 booster intervals. Twitter users in this study demonstrated a low understanding of the dose guidelines and how they should schedule their boosters after getting infected:

Some thoughts about vaccination. How do you do it if you just had COVID-19, do you take booster 3 or should you wait a couple of months?

Some tweets were very specific in that they asked questions related to certain medical conditions or age groups:

Look at the risk to the foetus/mother. These are extremely low if the mother is healthy, not overweight...How much risk should COVID-19 pose to recommend a vaccine where the clinical studies are not complete?

## Discussion

### Principal Findings

The overall aim of the study was to understand the attitudes of Swedish Twitter users toward COVID-19 vaccines. The study found that tweets expressing opinions about vaccines and the vaccination process were predominantly negative. The tweets expressed low confidence in the COVID-19 vaccines, policy makers, and scientific experts. Further concerns were related to complacency, which reflected a low understanding of the severity of COVID-19 infection and a low perception of the necessity to vaccinate, particularly with regard to booster shots. Moreover, the study found that convenience was not seen as a major challenge; however, the accessibility of information and the quality and availability of vaccination services were criticized.

Swedish Twitter users in this study had major concerns about the safety of the vaccines. This supports the findings of other studies that highlight the importance of people’s confidence in vaccine safety in promoting vaccination uptake [13,14,38]. Moreover, the results indicate the presence of rumors about the vaccine. For example, the fear of acquiring AIDS from vaccines could have undermined people’s willingness to be vaccinated, and there have been numerous studies that have highlighted rumors and misinformation about COVID-19 vaccines [5,39].

This study further demonstrates that people’s beliefs in the effectiveness of COVID-19 vaccines have decreased over time. The findings show that these arguments were commonly raised against COVID-19 vaccines and booster shot uptakes and might be related to the low booster coverage seen among the Swedish population [5].

The data further show that people express mistrust in authorities and demand more transparency from the government and the responsible authorities, mainly from the Public Health Agency of Sweden and the Swedish Medical Products Agency (SMPA), about the incidence and severity of the side effects caused by the vaccines. The SMPA releases a monthly update of the registered side effects and a list of death cases suspected to be related to COVID-19 vaccines [40,41]. This indicates that the information was available but might not have been effectively shared with the public. The mistrust in the government and health authorities’ management of the COVID-19 pandemic found in this study is a new finding and is not consistent with previous studies in the field. A survey from 2020 found that most of the Swedish population supported the government’s strategy in managing the pandemic and had strong trust in health authorities [42]. However, these findings do not necessarily reflect the opinion of the population today; in fact, the findings from our study indicated that trust in the authority’s management might have been negatively affected by the continuous changes in guidelines for taking booster shots.

Tweets analyzed under the *complacency* theme suggested that the perceived severity of COVID-19 infection was low, and consequently, that the perceived importance of the vaccine has been fading. This may be largely because of rumors and limited knowledge of vaccines. This study shows a widespread belief in the superiority of natural immunity and the low risk of COVID-19 infection in healthy individuals. The data also show that people were actively encouraging others to get infected rather than get vaccinated. These results are consistent with a Portuguese study, which documented that a low perceived risk among healthy adults contributes to their vaccine hesitancy [38]. The SMPA and the Centers for Disease Control and Prevention warned against these beliefs and emphasized that the risks associated with COVID-19 infection are greater than the risks of taking the vaccine, and that the immune response against the infection is not foreseeable; therefore, no groups are protected from becoming seriously ill [43,44]. This study further found conflicting views on the risks and benefits of vaccinating children against COVID-19. Users presented opposing evidence regarding the risk of infection and safety of vaccines for children. These findings are consistent with the Public Health Agency of Sweden’s survey results, which highlight the uncertainty among parents regarding their children’s vaccination [45].

The *convenience* concerns expressed among the tweets were related to limited access to and availability of vaccination appointments. The findings implied that although drop-in vaccination services were introduced, better organization shortened the queue times. These results suggest that enhancing the efficiency of vaccination services could encourage vaccination. Furthermore, this study reveals that there was a lack of knowledge about COVID-19 and the vaccines, which could arguably have resulted in people turning to social media to seek answers to their unanswered questions. This aligns with a survey conducted in 2020 that showed that part of the Swedish population was not satisfied with the information provided by health authorities on COVID-19 [42]. There is growing evidence of a lack of information causing hesitancy. For instance, a study conducted in the United States before the development of COVID-19 vaccines found that people were willing to get vaccinated if they received adequate information about the vaccines [46], whereas another study on the influenza vaccine found that those with better influenza literacy had higher chances of choosing vaccination [47].

### Limitations

This study has some limitations. It is important to note that Twitter API provides free access to only a 1% sample of all Twitter data [48], thus limiting the generalization of the findings. In addition, the study timeline was limited to 2 months, which does not represent the general perspective of the web-based population. Longer studies would strengthen the validity of these results.

In addition, although the number of web-based users in Sweden has increased in recent years, they cannot be considered representative of the entire Swedish population. As it was not possible to capture users’ demographics for technical reasons, as such details are not available on Twitter, the transferability of the study is also limited because of the lack of such information. However, studies on the demographics of Twitter users have shown that the web-based population constitutes the younger generation, with females especially overrepresented [49]. Furthermore, the study’s results and parameters are specific to the Swedish context; thus, a similar study in other settings could present different concerns and opinions.

Finally, there are limitations to language-restricted searches as they may include Swedish expats who live in a different context than Sweden. It is of course possible that expats are part of the discussion; according to this report, as many as 700,000 Swedes actually live abroad, or around 7% of Swedish people [50]. However, as they are a relatively small proportion of the total population, we doubt that they heavily skew our findings.

### Public Health and Practical Implications

This study contributes to ongoing public health efforts to promote COVID-19 vaccination and address vaccine hesitancy, particularly in Sweden. These data were collected in early 2022, when COVID-19 vaccine coverage for 2 doses was nearly 90% of the eligible Swedish population. Since then, Sweden has experienced a decrease in the uptake of the third dose [5]. Weaning confidence in booster vaccines was observed in this study. This highlights the importance of monitoring and analyzing public sentiments regarding health-related matters, particularly vaccine decision-making, on social media platforms. As the number of social media users is rapidly increasing, the social media landscape has emerged as an important platform that must be considered when working with public health awareness. Moreover, this study provides evidence of the dominance of negative attitudes on social media, which forms a threat to public health and needs to be addressed.

This study indicates that COVID-19 vaccine acceptance is not unchallenged and should be closely monitored to address emerging COVID-19 vaccine hesitancy. It also shows that social media studies provide valuable insights into the factors that shape public attitudes toward vaccination. Furthermore, the evidence illustrates that clear health communication and consistent messages are needed to maintain public trust. Moreover, this study emphasizes the importance of addressing the spread of rumors and misinformation to overcome COVID-19 vaccine hesitancy and its potential implications for future vaccines.

This study contributes to the existing literature on COVID-19 vaccines by exploring the attitudes of Swedish users on the web. Still, further social media studies are needed to explore and quantify attitudes toward COVID-19 vaccines on the entire spectrum of social media platforms.

Public trust in government, experts, and authorities can be reinforced by facilitating open dialogue and channels with the public. Furthermore, innovative approaches, such as internet-based interventions to address the growing web-based community, could be considered to increase public trust in COVID-19 vaccines. The Public Health Agency of Sweden and other health-related authorities should expand their presence on the web to provide accurate information on various social media platforms. Additional resources should be considered to increase the quality of vaccination services and the vaccination support system to provide opportunities for personalized consultations on vaccination.

### Conclusions

This study shows that Swedish Twitter users engaged in discussing COVID-19 vaccination expressed safety and effectiveness concerns about COVID-19 vaccines and mistrust in governmental authorities, scientific organizations, and media agencies. The tweets indicated a fading belief in vaccination necessity linked to changes in the perceived severity of COVID-19 infection and belief in the superiority of natural immunity. In comparison, the quality of vaccination services was discussed less frequently; however, some complaints related to the limited availability of vaccination appointments did appear. Moreover, there was an observed information gap on COVID-19 and vaccines related to contradicting evidence and unanswered questions. The study highlights the importance of enhancing health communication, increasing public trust in the government, and countering misinformation.

## Abbreviations

API: application programming interface
SMPA: Swedish Medical Products Agency
TA: thematic analysis
WHO: World Health Organization
